# Case Report: A novel *TP53* mutation in a patient with quadruple wild-type gastrointestinal stromal tumor

**DOI:** 10.3389/fonc.2023.1260706

**Published:** 2023-11-09

**Authors:** Yuhong Chen, Junyong Chen, Liansheng Long, Leng Han, Xiaohui Mi, Yanfang Song, Huanqing Cheng, Yanrui Zhang, Liyang Cheng

**Affiliations:** ^1^ Department of General Surgery, the General Hospital of Southern Theater Command, People’s Liberation Army (PLA), Guangzhou, China; ^2^ Department of Pathology, The General Hospital of Southern Theater Command, People’s Liberation Army (PLA), Guangzhou, China; ^3^ Medical Affairs Department, Acornmed Biotechnology Co., Ltd, Beijing, China

**Keywords:** case report, quadruple WT GIST, next-generation sequencing, *TP53* mutation, p53-targeted therapy

## Abstract

In this report, we present a case study of a 64-year-old female who was diagnosed with gastrointestinal stromal tumors (GISTs) and subsequently developed liver metastases despite undergoing radical resection. Next-generation sequencing (NGS) assays indicated that the tumor lacked *KIT/PDGFRA/SDH/RAS-P* (*RAS* pathways, *RAS-P*) mutations, thereby classifying this patient as quadruple WT GIST (qGIST). Treatment with imatinib was initiated, and after 2.5 months, recurrence of the tumor and multiple metastases around the surgical site were observed. Consequently, the patient was switched to sunitinib treatment and responded well. Although she responded well to sunitinib, the patient died of tumor dissemination within 4 months. This case study highlights the potential efficacy of imatinib and the VEGFR-TKI sunitinib in treating qGIST patients harboring a *TP53* missense mutation.

## Introduction

Gastrointestinal stromal tumors (GISTs) are the most common of all sarcomas (1). Most GISTs occur in the stomach (60–65%), and the second most common site is the small intestine (20–25%); the rectum, colon, esophagus, and other sites are rare (1, 2). GISTs are a heterogeneous group of tumors that includes a variety of molecular entities. Approximately 85-90% of GISTs harbor *KIT* or *PDGFRA* mutations, and the remaining GISTs that do not harbor any mutation of the *KIT* and *PDGFRA* genes are defined as wild-type (WT) GISTs. Among WT GISTs, 20-40% of tumors are *SDH*-deficient GISTs that show loss of function of the succinate dehydrogenase complex (SDH). Approximately 15% of *KIT/PDGFRA* WT cases harbor mutations in *BRAF/RAS* or *NF1* and are referred to as RAS-pathway (RAS-P) mutant GISTs. In addition, a small subset of all GISTs (5%) that lack *KIT/PDGFRA/SDH/RAS-P* (*RAS* pathways, *RAS-P*) mutations can be referred to as quadruple WT GISTs or quadruple negative GISTs (3). To date, a variety of sporadic somatic molecular events in quadruple WT GISTs have been reported, including *TP53, MEN1, MAX, CHD4, FGFR1, CTDNN2, CBL, ARID1A, BCOR, and APC* (4).

*TP53* is a critical tumor suppressor gene that encodes a 64 kDa protein (5, 6). Mutations in *TP53* not only impair its antitumor activity but also confer mutant p53 protein oncogenic properties. *TP53* gene mutations are found in a variety of tumors (6). However, information about mutations in *TP53* that occur in quadruple WT GISTs is limited. Recently, the frequency of mutant *TP53* in GISTs was reported to be 3.5%, and only one quadruple WT GIST case harbored a *TP53* mutation (p.V172F) (5). In addition, approaches for p53-targeted therapy in quadruple WT GISTs are rare.

To our knowledge, imatinib, which targets KIT/PDGFRA, has been used as a standard first-line treatment for patients with localized and advanced GISTs (7). However, 50-60% of patients with GISTs may exhibit primary or secondary resistance to imatinib (8). In particular, WT GIST is known to be generally unresponsive to the tyrosine kinase inhibitor imatinib used for non-WT GIST (9, 10). In addition, there is no consensus on the treatment of qGIST owing to the variety of molecular subtypes. Thus, identifying gene mutations in different patients is necessary to guide therapy and improve prognosis by matching targeted drugs.

Here, we present a quadruple WT GIST case that harbored a *TP53* missense mutation identified by next-generation sequencing. The patient progressed rapidly after imatinib treatment and subsequently achieved remission with the VEGFR-TKI sunitinib, which is a p53-targeted therapy. This case provides new insights into the rare treatment of qGIST.

## Case presentation

**Figure 1:**
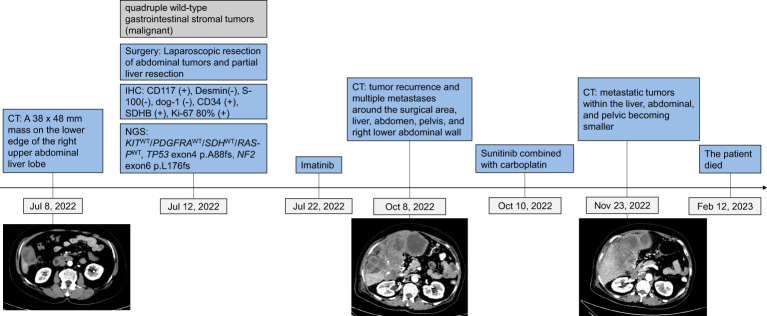
Tumor progression and treatment of a patient with quadruple wild-type gastrointestinal stromal tumors.

A 64-year-old female presented at Southern Theater General Hospital for right abdominal pain that had persisted for over 3 months. Computed tomography (CT) imaging on July 8, 2022, showed a mass on the lower edge of the right upper abdominal liver lobe approximately 33 X 48 mm, and the boundary was unclear (**Figure 1**). The adjacent peritoneum, colon and liver were not clearly demarcated, the adjacent peritoneum was slightly thickened, its internal density was uneven, and it seemed to have punctate calcification foci and a slightly irregular low density. Thus, the CT result indicated the possibility of colon exophytic malignant stromal tumors. The tumor and part of the liver were surgically removed in July 2022 (**Figure 1**).

**Figure 2:**
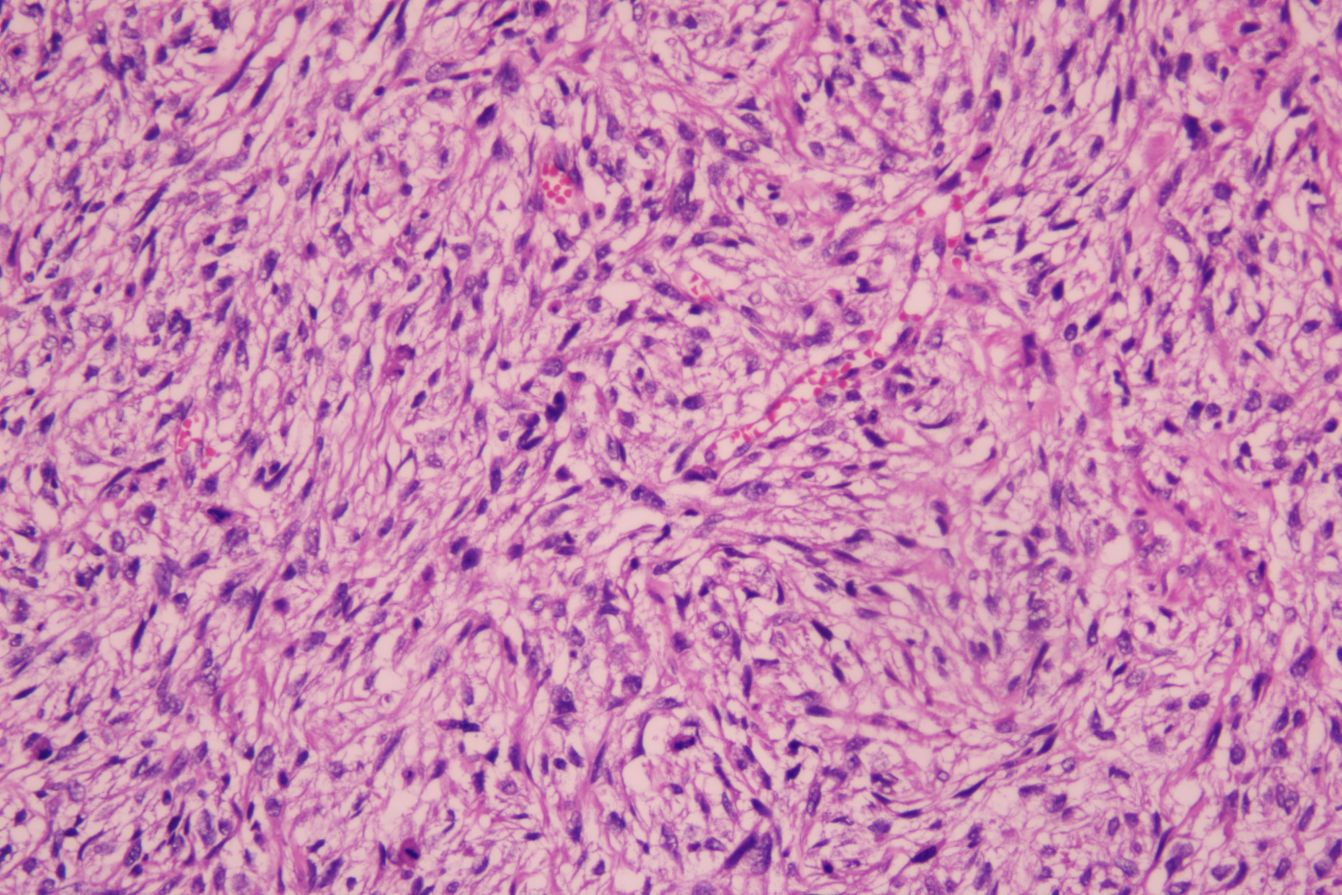
Photomicrograph of gastrointestinal stromal tumor (H&E, x200).

Histopathological examination of the excised mass showed spindle cells and epithelioid cells were mixed. The spindle cells were arranged in bundles, in a braided and palisade manner, with varying cell density, no significant pleomorphism, and visible nuclear vacuoles. Epithelioid cells were distributed in sheets with clear cell boundaries. Multinucleated giant cells were observed in some areas and mitotic images could be seen. Fibrous demarcation was observed between the spindle cells and epithelioid cells. Focal stroma exhibited myxoid alteration (**Figure 2**).

**Figure 3:**
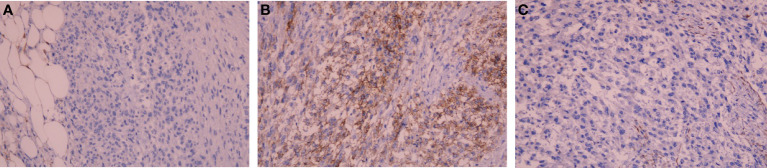
Photomicrograph showing negative immunohistochemical stains of **(A)** Desmin and **(B)** S-100, positive immunohistochemical stains of **(C)** CD117 (IHC x200).

On immunohistochemistry, the tumor cells were positive for CD117, negative for Desmin and S-100 (**Figure 3**), negative for dog-1, positive for CD34, and positive for SDHB. The Ki-67 labeling index was 80%. The mutation status of the tissue sample was detected by using the Next-generation sequencing (NGS)-based Acorn808 gene panel (Acornmed, Beijing, China), which covers all the coding exons of 808 tumor-related genes that are frequently mutated in solid tumors. The NGS results revealed no mutations in the exons corresponding to the *c-KIT* and *PDGFRA* genes, including exons 9, 11, 13 and 17 of *KIT* and exons 12, 14 and 18 of *PDGFRA*. This patient lacked mutations in *RAS-P (NF-1, BRAF, RAS)*. Therefore, this GIST patient had quadruple WT GIST (*KIT* ^WT^/*PDGFRA* ^WT^/*SDH* ^WT^/*RAS-P* ^WT^). Interestingly, NGS revealed the presence of a T*P53* exon 4 p. A88fs (81.56% abundance in tissue) and *NF2* exon 5 p. L176fs (79.53% abundance in tissue) somatic mutation (**Table 1**).

**Table 1:** Summary of gene test results and mutations that may have clinical significance.

Gene	Exon	Mutation	Variation Frequency
*TP53*	exon 4	A88fs	81.56%
*NF2*	exon 6	L176fs	79.53%
*CDKN2A*	–	copy number loss	0.41

According to the NCCN guidelines Version 2. 2022 Gastrointestinal stromal tumors (GISTs), TKIs could offer a treatment option with relatively good responses for wild-type GISTs. Thus, postoperative adjuvant imatinib therapy was recommended for this patient. The patient received imatinib (400 mg/day) treatment from July 22, 2022, to October 7, 2022. The CT result on October 8, 2022, indicated tumor recurrence and multiple metastases around the surgical area, liver, abdomen, pelvis, and right lower abdominal wall, with a lesion size of 14*11*8 cm in the liver area.

Considering that sunitib is the standard second-line therapy for GISTs, and *TP53* exon 4 p. A88fs indicates that patients with solid tumors might be sensitive to VEGFR-TKIs (11), we treated the patient with sunitinib (2 weeks of treatment at a dose of 37.5 mg/day and 1 week of rest) in combination with carboplatin (AUC 6, IV, d1, q21d). The patient responded well to sunitinib at first, with metastatic tumors in the liver, abdominal, and pelvic becoming smaller and a lesion in the liver region measuring 10*8*7 cm; however, she died of tumor dissemination on February 12, 2023.

## Discussion

Here, we show a rare quadruple WT GIST with *TP53* mutation. This quadruple WT GIST patient’s disease progressed rapidly after initial imatinib treatment; however, after switching to sunitinib, the disease responded well. GISTs are uncommon neoplasms that originate from the interstitial cells of Cajal located within the gastrointestinal tract. GISTs are commonly present in the stomach (60-65% of cases), followed by the small intestine (20-25%), while occurrence in other locations, such as the rectum, colon, and esophagus, is infrequent (1). In this case, the tumor originated from the abdominal region, with its location potentially impacting the patient’s prognosis, which has rarely been reported. *KIT* and *PDGFRA* mutations are the most common mutations found in GISTs, accounting for approximately 85-90% of cases. There is a rare subtype of GISTs that do not harbor any mutations in the *KIT, PDGFRA, SDH, and RAS-P* genes, and these are defined as quadruple WT GISTs (1, 3).

*TP53* gene mutations have been found in a small percentage of GISTs, particularly in the quadruple WT subgroup. The *TP53* gene, also known as tumor protein 53, is a well-known tumor suppressor gene that is frequently mutated in many different types of cancer (4). At present, the exact role of *TP53* mutations in the development and progression of GISTs remains unclear. However, it is believed that *TP53* mutations may play a role in the development of GISTs by promoting genomic instability and thereby facilitating the acquisition of additional genetic alterations that contribute to tumor development and progression (11). Studies have suggested that *TP53* mutations may be associated with poorer prognosis and decreased survival in patients with GISTs (6). Additionally, preclinical studies have suggested that *TP53* mutations may confer resistance to certain therapies, such as imatinib, which is a standard treatment for GISTs (5). While *TP53* mutations are not common in GISTs, their association with qWT GISTs and possible involvement in tumorigenesis and treatment resistance merits further investigation.

Although imatinib treatment for GIST has been recognized as the model of precision oncology, there is still substantial confusion on how to manage quadruple WT GISTs (2). The discovery of the *TP53* mutation and *CDKN2A* copy number loss may be the reason for the patient’s rapid disease progression following imatinib therapy. The poor prognostic effect of *TP53* gene overexpression and its association with higher malignant risk in GIST have already been described (11). As is common knowledge, the *TP53* gene plays a role in the activation of DNA repair and apoptosis initiation. For several decades, the role of TP53 dysregulation in carcinogenesis has been explored in human tumors and linked to both tumor-suppressing and oncogenic function loss (12).Tumors with p53 mutations often develop more rapidly, respond poorly to anticancer therapy and have a poor prognosis (5, 6). Another possibly relevant mechanism of GIST progression toward more aggressive biological behavior is a partial deletion of chromosome 9, which plays an important role in the transition from low- to high-risk GIST (5). The loss of the cell cycle inhibitor p16 (CDKN2A), which is a result of this deletion, is one potential outcome. Inactivating mutations in this gene or its deletion appear to be required to increase cell cycle activity and, as a result, progress from low- to high-risk GIST (5).

Imatinib is the standard treatment for GIST, however, it is already known that the absence of *KIT* or *PDGFRA* mutations confers greater resistance to imatinib (10, 13). In addition, preclinical studies have described that *TP53* mutations in GIST may not be sensitive to imatinib treatment (5). It appears that most KIT/PDGFRA WT GISTs have an indolent clinical behavior that is not predicted by conventional risk stratification (13). And the patient underwent rapid progression after standard treatment with imatinib, suggesting that imatinib may not be suitable as a first-line treatment for qGIST. Considering sunitinib as a standard second line treatment and NGS detection of *TP53* mutation, the patient received treatment with sunitinib, with a significant remission. Currently, it has been documented that patients with *TP53* mutations potentially respond significantly to VEGFR TKI therapy (11). However, up to 50-60% of GISTs show primary or secondary resistance to imatinib (8). Advanced resistant GIST responds to second-line sunitinib, third-line regorafenib, and the novel broad-spectrum TKI ripretinib (1, 8). Combined with the gene test results, the patient was given sunitinib after first-line imatinib resistance. Encouragingly, the patient initially responded well with smaller metastatic tumors in the liver, abdomen and pelvis than before, and the size of the lesion in the liver region was reduced to 10*8*7 cm after sunitinib treatment (the size of the tumor in the liver metastasis after imatinib treatment was 14*11*8 cm). It may be worthwhile to use sunitinib as a first-line treatment option for patients with qGIST. Unfortunately, the patient succumbed to tumor dissemination, which was possibly related to sunitinib resistance after remission, highlighting the need for more careful monitoring and treatment. Recent studies have shown that the p53 pathway may be involved in the resistance of renal cell carcinoma cells to sunitinib, and that p53-positive cases tend to be associated with poor progression-free survival (PFS) after first-line sunitinib treatment (14). A clinical trial study (IMmotion151) also showed *TP53* mutation was significantly associated with poor PFS in metastatic RCC (mRCC) treated with first-line sunitinib (14). Further research is evidently required to delve into qGIST, especially in combination with *TP53* mutation.

## Conclusions

In conclusion, we reported a case of a 64-year-old female with GIST who developed liver metastases despite surgery. Genetic testing revealed that the tumor lacked specific mutations, classifying it as quadruple qGIST. While treated with imatinib, the tumor recurred, but the patient responded well to sunitinib. However, she passed away due to tumor dissemination within four months. This case suggests that qGIST patients with *TP53* mutations may benefit from treatment with the VEGFR-TKI sunitinib, but further research is needed to optimize treatment strategies.

## Data availability statement

The original contributions presented in the study are included in the article/supplementary material. Further inquiries can be directed to the corresponding author.

## Ethics statement

Written informed consent was obtained from the individual(s) for the publication of any potentially identifiable images or data included in this article.

## Author contributions

LC: Funding acquisition, Supervision, Writing – review and editing, Conceptualization, Resources. YC: Writing – original draft, Investigation. JC: Writing – review and editing, Data curation. LL: Writing – review and editing, Methodology. XM: Formal Analysis, Project administration, Software, Writing – review and editing. YS: Formal Analysis, Software, Writing – review and editing. YZ: Formal Analysis, Software, Writing – review and editing. HC: Writing – review & editing, LH: Data curation.
